# NOTCH3 inhibits transcription factor ZEB1 expression and metastasis of breast cancer cells via transcriptionally upregulating miR-223

**DOI:** 10.7150/jca.89034

**Published:** 2024-01-01

**Authors:** Wen-Jia Chen, Hui-Ting Zhong, Hua-Tao Wu, Yan-Yu Hou, Zheng Wu, Ze-Xuan Fang, Jing Liu

**Affiliations:** 1 The Breast Cancer, Cancer Hospital of Shantou University Medical College, Shantou 515041, China.; 2 Department of Physiology/Changjiang Scholar's Laboratory, Shantou University Medical College, Shantou 515041, China.; 3 Department of Breast Surgery, Huizhou Municipal Central Hospital, Huizhou 516000, China.; 4 Department of General Surgery, First Affiliated Hospital of Shantou University Medical College, Shantou 515041, China.

**Keywords:** NOTCH3, miR-223, ZEB1, breast cancer, metastasis

## Abstract

**Background:** NOTCH receptor 3 (NOTCH3) and zinc finger E-box binding protein 1 (ZEB1) play important roles in breast cancer respectively. NOTCH3 maintains the luminal phenotype and inhibits epithelial-mesenchymal transition (EMT) in breast cancer, while ZEB1 and NOTCH3 have the opposite effects.

**Methods:** Public databases were used to predict the expression of NOTCH3 and ZEB1 in breast cancer cell lines. The regulatory effect of NOTCH3 on ZEB1 expression was verified by western blot and RT-PCR. MiRNAs regulating ZEB1 expression were identified by using multiple databases and confirmed by reporter gene experiments. Cellular function experiments were conducted to evaluate the role of NOTCH3/miR-223/ZEB1 in the proliferation and invasion of triple-negative breast cancer (TNBC).

**Results:** NOTCH3 and ZEB1 have opposite expression pattern in MCF-7 cells that over-express LncATB or were incubated in TGF-β to induce EMT. Western blotting and RT-PCR showed that NOTCH3 could regulate expression of ZEB1. MiR-223 inhibited the proliferation and invasion of breast cancer cells via down-regulating the expression of ZEB1. NOTCH3 inhibited the proliferation and invasion of breast cancer cells via up-regulating the expression of miR-223. Clinically, high expression of NOTCH3, miR-223 or low expression of ZEB1 were related to good prognosis of breast cancer patients.

**Conclusion:** The current study reports a novel NOTCH3/miR-223/ZEB1 axis, which can inhibit the proliferation and invasion of breast cancer cells, and may serve as a potential biomarker for the prognosis of breast cancer.

## 1. Introduction

Breast cancer is a major challenge to global health. In 2020, it became the most commonly diagnosed cancer and the main cause of cancer death in women worldwide 1. Metastasis is the main cause of treatment failure and death of most patients. Therefore, the prognosis of patients is closely related to metastasis 2. Metastasis is a complicated process involving many cellular mechanisms, including cell division, invasion, escape from immune surveillance and changes in tissue microenvironment, especially epithelial-mesenchymal transition (EMT), which is necessary for most cancers to metastasize 3. Among different molecular subtypes, triple-negative breast cancer (TNBC) is highly aggressive, has a high metastasis rate and confers poor prognosis 4. Recently, various FDA-approved drugs for metastatic breast cancer, including doxorubicin, cyclophosphamide and vorinostat, have many limitations 5-7. Therefore, there is an urgent need to find new biomarkers to alert breast cancer metastasis and explore potential molecular mechanisms to develop potential treatment strategies to improve the survival and prognosis of breast cancer patients.

NOTCH is a highly conserved signaling pathway that is involved in many biological processes, including stem cell self-renewal, cell differentiation, proliferation, migration, adhesion, survival, and apoptosis 8, and abnormal NOTCH signaling has been linked to a variety of human diseases, including malignant transformation of the breast 9, 10. In the NOTCH signaling pathway, when a ligand (DLL1、DLL3、DLL4、Jagged1 and Jagged2) binds to a NOTCH receptor (NOTCH1-4), it induces the release and nuclear translocation of NOTCH receptor intracellular domain (NICD), which interacts with the transcription factor CSL, leading to the transcriptional activation of its target genes. Different from the oncogenic role of NOTCH1/2/4 in breast cancer, which promotes the transformation of ductal carcinoma in situ to invasive breast cancer 11-13, NOTCH3 is considered to be a tumor suppressor in breast cancer 14, 15. In breast cancer cells, NOTCH3 can activate the Hippo/YAP pathway by up-regulating Kibra, thereby inhibiting EMT 16, and NOTCH3 inhibits EMT in breast cancer by transcriptionally up-regulating the expression of GATA-3 17, suggesting that NOTCH3 inhibits the progression of breast cancer mainly through transcriptional activation of downstream target genes.

Another transcription factor ZEB1, which binds to E-box motifs in the promoter of downstream genes and is reported to repress E-cadherin transcription in breast cancer, is also a key factor in EMT 18. ZEB1 is expressed in various tissues, including bone 19, smooth muscle 20, and nerve 21. ZEB1 is abnormally expressed in various human cancers, including pancreatic cancer 22, lung cancer 23, liver cancer 24, colon cancer 25, and breast cancer 26, 27. ZEB1 plays a key role in tumor progression, metastasis, invasion and treatment resistance 28, 29, as well as in regulating the differentiation and metastasis of breast cancer 30. Our preliminary results showed the opposite expression pattern of NOTCH3 and ZEB1 in breast cancer, which predicts a potential regulatory axis between NOTCH3 and ZEB1. The current study aims to evaluate the correlation between NOTCH3 and ZEB1 and investigate the potential regulatory axis in breast cancer.

## 2. Materials and methods

### 2.1 Cell culture

Human breast cancer cell lines MCF-7 and MDA-MB-231 cells were obtained from the American Type Culture Collection (ATCC). All cells were cultured in DMEM (Invitrogen, CA, USA) supplemented with 10% fetal bovine serum (FBS) and 1% penicillin/streptomycin (Invitrogen, CA, USA) at 37°C in a 5% CO2 incubator.

### 2.2 Transfection and reagents

The plasmid pCMV-Sport6-N3ICD and its vector pCMV-Sport6 were gifts from Prof. Michael M. Wang (University of Michigan, USA) 31, while pcDNA3.1(+)-lncATB was described previously 32. Human ZEB1 cDNAs were cloned into RP-EGFP/puro, pmirGLO-ZEB1-3'UTR and pmirGLO-ZEB1-3'UTR-Mut were constructed in the pmirGLO vector, and pGL3-miR-223-pro and pGL3-miR-223-pro-Mut were constructed in pGL3-Enhancer. The miR-223 mimics and inhibitors, specific siRNAs targeting NOTCH3 and ZEB1, as well as control siRNAs, were designed and synthesized by GenePharma (Suzhou, China). The siRNA sequences are listed in Table 1. Transient transfection was performed using Lipofectamine 3000 (Thermo Fisher, MA, USA). After 48 hours of transfection, RNA or protein was extracted to determine transfection efficiency.

### 2.3 Reverse transcription and PCR analysis

Total RNA was isolated from cells using Trizol Total RNA Isolation Reagent (Invitrogen, CA, USA) following the manufacturer's instructions and stored at -80 °C. Reverse transcription was performed using PrimeScript RT reagent kits DRR036A and DRR047A (TAKARA, Japan) according to the manufacturer's instructions. qRT-PCR was performed with SYBR Select Master Mix (Thermo Fisher, MA, USA) on a CFX96 Real-time PCR Detection System (Bio-Rad, CA, USA) to measure the expression levels of mRNAs and miRNAs. Primer sequences for qRT-PCR are listed in Table 2 and Supplementary Table S1.

### 2.4 Western blot analysis

Cellular whole protein was extracted with RIPA lysis buffer (Millipore, USA), analyzed by western blotting and visualized on ChemiDoc XRS+ (Bio-Rad, USA). Briefly, cells were lysed in RIPA buffer with 1 mM phenylmethylsulfonyl fluoride and phosphatase inhibitors (5 mM sodium orthovanadate). Protein lysates were separated by SDS-PAGE, transferred to a PVDF membrane, and immunoblotted with primary antibodies at 4 °C overnight. Antibodies used and volume dilution were listed in Table 3.

### 2.5 Cell proliferation assay

We seeded 1.0×10^3^ MDA-MB-231 cells in each well of a 96-well microplate. Proliferation was measured using a Cell Counting Kit-8 (Beyotime Biotechnology). Absorbance was measured at 450 nm with a microplate reader ELX800 (Bio-Tek, Winooski, VT, USA).

### 2.6 Colony-formation assay

A total of 1.0×10^3^ MDA-MB-231 cells or 1.5×10^3^ MCF-7 cells were seeded in each well of a six-well plate. After 2 weeks, cells were fixed with methyl alcohol and stained with 0.1% crystal violet, and the number of colonies was counted under a Zeiss microscope (Zeiss, Oberkochen, Germany).

### 2.7 Wound healing assay

MDA-MB-231 cells were plated in six-well plates and allowed to reach 95% confluence. After starvation by depriving serum for 24 h, a linear wound was created using a 200 μL tip. After rinsing with phosphate-buffered saline, cells were cultured with FBS-free medium (Fetal Bovine Serum) and allowed to migrate. Photographs were taken (×40) after 24 h (MDA-MB-231). Wound healing was photographed every 24 hours.

### 2.8 Transwell assay

Cell culture inserts (8 μM pore size; BD, Franklin Lakes, NJ, USA) and Matrigel invasion chambers 33 were used to perform migration and invasion assays, respectively. After serum-starvation for 24 h, 2.0×10^4^ MDA-MB-231 cells, suspended in serum-free medium, were seeded into the upper chamber, while the bottom chamber contained complete medium. For the migration assay, 24 h (MDA-MB-231) later, non-migrated cells from the upper side of the chamber were removed, and cells on the lower side of the chambers were fixed in methanol and stained with 0.1% crystal violet. For the invasion assay, 36 h (MDA-MB-231) later, invaded cells were collected. The rest of the protocol was similar to the cell migration assays. Each experiment was carried out in triplicate. The exact number of cells from 5 random fields in every individual well was captured and calculated by two investigators.

### 2.9 Dual-luciferase reporter assay

To investigate the effect of miR-223 on ZEB1-3'UTR activities, and NOTCH3 on the promoter of miR-223, dual-luciferase reporter assays were performed with their reporter vector and pRL-SV40 (Promega). After transfection with a miR-223 mimic/inhibitor or NOTCH3 plasmid, luciferase activities were measured with a Dual-Luciferase Assay kit (E1910, Promega). The relative luciferase activity was calculated as the ratio of firefly to Renilla luciferase activities.

### 2.10 Online data acquisition and analysis

The Broad Institute Cancer Cell Line Encyclopedia (CCLE, https://sites.broadinstitute.org/ccle/) and The Human Protein Atlas (THPA, https://www.proteinatlas.org/) databases were used to search and obtain the expression level of NOTCH3 and ZEB1 in different breast cancer cell lines. The CCLE databases were also used to search and obtain the expression level of miR-223 in different breast cancer cell lines. Potential miRNA binding to the 3' UTR of ZEB1 was predicted by TargetScan 7.2 (http://www.targetscan.org/vert_72/), StarBase (http://starbase.sysu.edu.cn/starbase2/index.php) and miRcode (http://mircode.org/). The promoter region of miR-223 was searched and downloaded from UCSC (http://genome.ucsc.edu/). Long-term Outcome and Gene Expression Profiling Database of pan-cancers (LOGpc, https://bioinfo.henu.edu.cn/DatabaseList.jsp) was used to predict the prognostic value of miR-223 in patients with breast cancer, while Kaplan-Meier Plotter (http://kmplot.com/) and PanCanSurvPlot (https://smuonco.shinyapps.io/PanCanSurvPlot/, GSE19615 and GSE26304 for OS, GSE20685 for MFS, GSE69032 for DRFS) were used for the prognostic value of miR-223, NOTCH3 and ZEB1 in patients with breast cancer.

### 2.11 Statistical analysis

Each experiment was repeated at least three times. Statistical analyses were performed using Student's *t* test and one-way ANOVA. Levels of statistical significance were evaluated with data using the chi-square test or Fisher's exact test for categorical variables. For all the analyses, *p* < 0.05 was considered statistically significant.

## 3. Results

### 3.1 Opposing expression of NOTCH3 and ZEB1 in breast cancers

**Figure 1:**
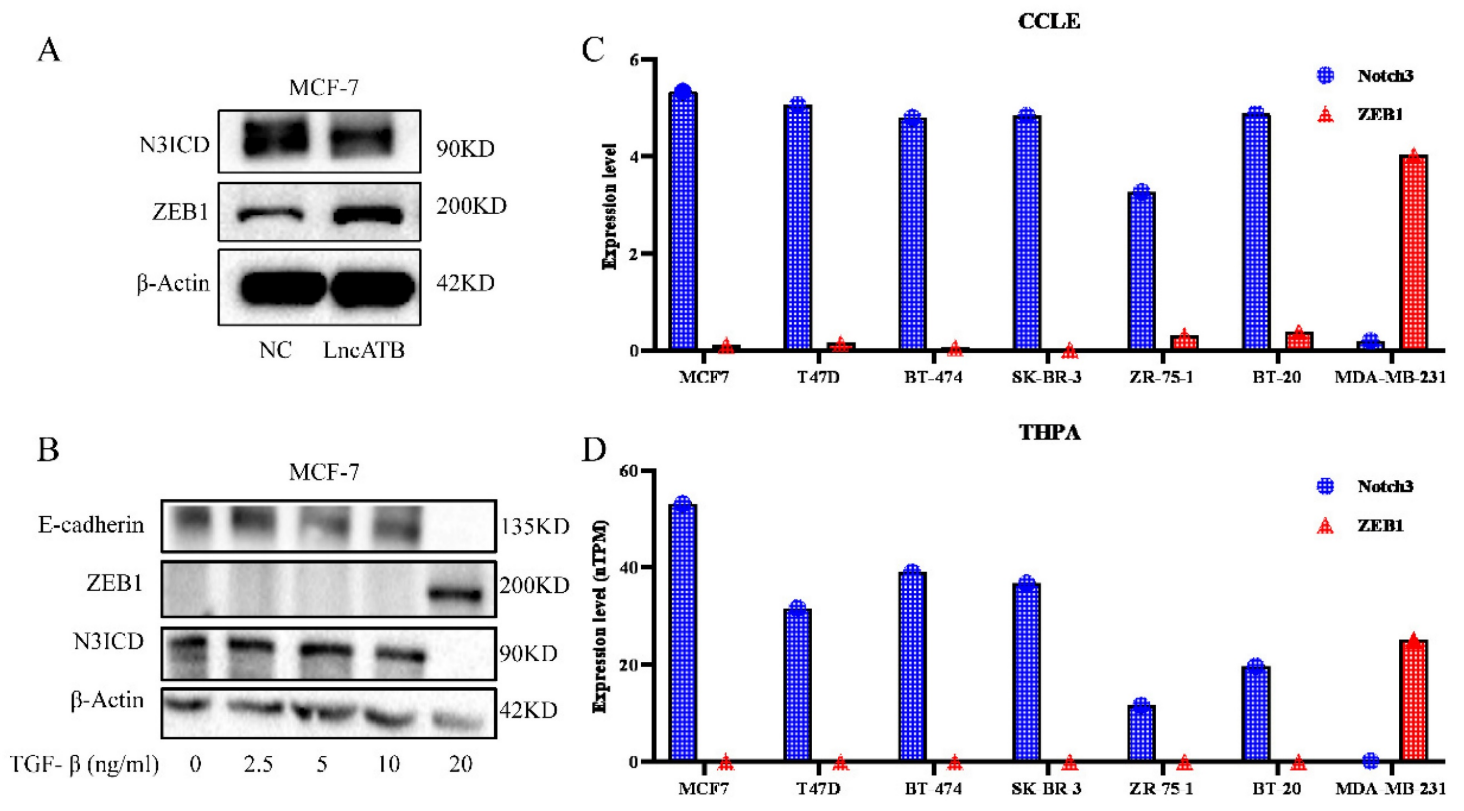
** Expression of NOTCH3 and ZEB1 in breast cancers.** (A) Western blot showing that after over-expression of LncATB in MCF-7 cells, expression of NOTCH3 decreased, while the expression of ZEB1 increased. (B) Western blot showing that the expression of E-cadherin and NOTCH3 decreased, while the expression of ZEB1 increased in MCF-7 cells treated with TGF-β. (C/D) CCLE and THPA databases showing the expression of NOTCH3 and ZEB1 in different breast cancer cell lines.

Our previous investigation reported that lncATB plays an oncogenic role in the development of breast cancer through regulating the miR-200c/Twist1 axis 34. Interestingly, in MCF7 cells overexpressing lncATB (Supplementary Figure S1), decreased NOTCH3 and increased ZEB1 levels were found (Figure 1A). We treated MCF-7 cells with different concentrations of TGF-β (0, 2.5, 5, 10, and 20 ng/ml) to induce EMT. Along with the increased TGF-β concentration, the expressions of E-cadherin and NOTCH3 decreased accordingly, while the expression of ZEB1 increased (Figure 1B).

In order to further explore the expression pattern of NOTCH3 and ZEB1 in breast cancer, online databases CCLE and THPA were mined to analyze the expression of NOTCH3 and ZEB1 in different breast cancer cell lines. It was found that the expressions of NOTCH3 and ZEB1 in breast cancer cells were negatively correlated (Figure 1C and 1D).

### 3.2 NOTCH3 suppresses the expression of ZEB1 in breast cancer cells

**Figure 2:**
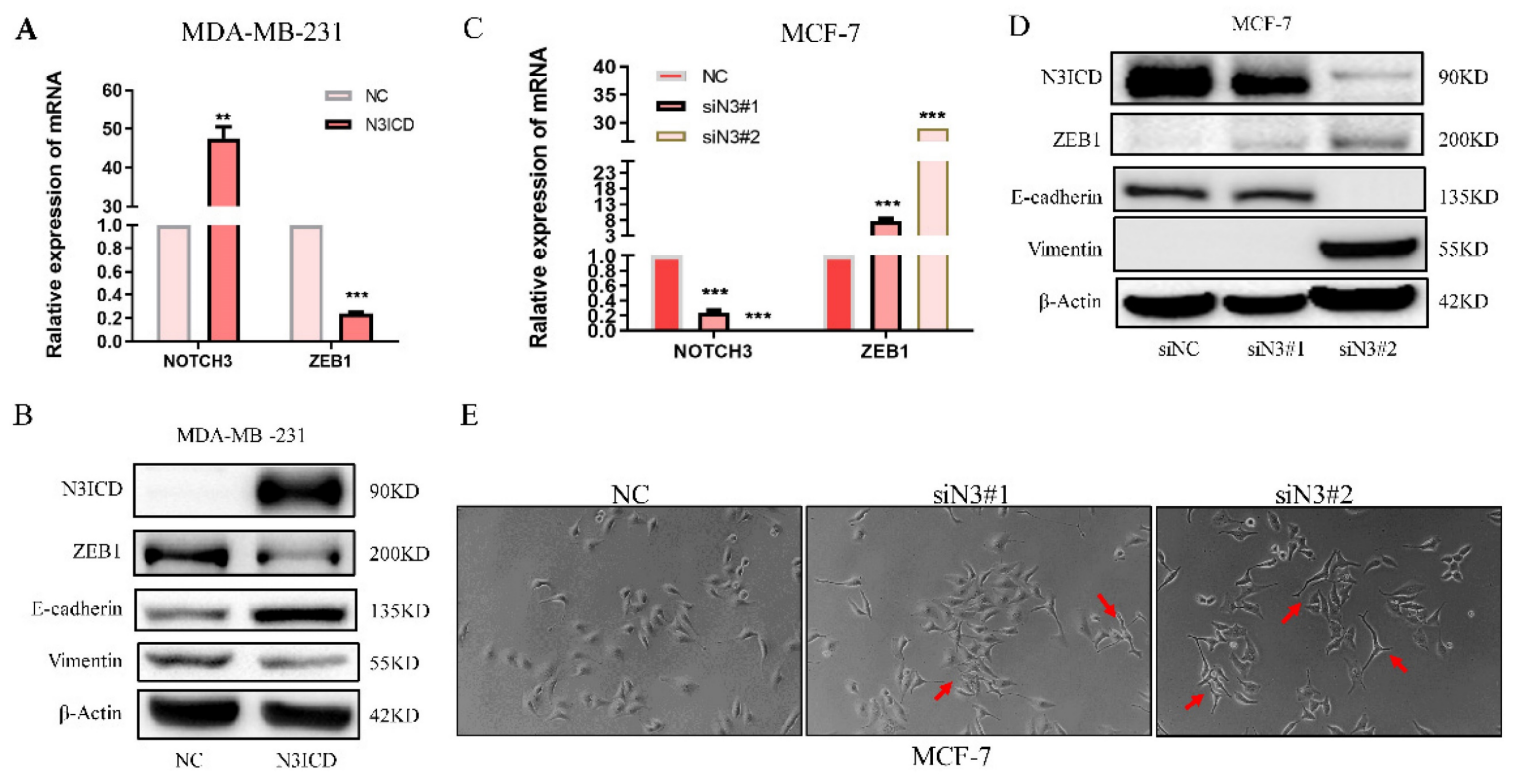
** NOTCH3 regulates the expression of ZEB1 in breast cancer cells.** (A/B) PCR and western blot results showing that after high overexpression of NOTCH3 in MDA-MB-231 cells, the expression of ZEB1 and vimentin decreased, while E-cadherin increased. (C/D) PCR and western blot assays showing that after NOTCH3 was knocked down in MCF-7 cells, the expression of ZEB1 and vimentin increased, while E-cadherin decreased. (E) The suppression of endogenous NOTCH3 in MCF-7 cells significantly changed their morphology with obvious pseudopodia stretching out (red arrows).

To explore the regulatory pattern between NOTCH3 and ZEB1, a NOTCH3 expression plasmid and siRNAs were transfected into MDA-MB-231 and MCF-7 cells. With increased NOTCH3, the mRNA and protein levels of ZEB1 were reduced accordingly (Figure 2A and 2B). The epithelial biomarker E-cadherin was increased, while the expression of vimentin, a mesenchymal biomarker, was decreased in N3ICD-overexpressing cells (Figure 2B). RNAi-mediated knockdown of endogenous NOTCH3, in MCF-7 cells, resulted in increased ZEB1 mRNA and protein, as well as decreased E-cadherin and increased vimentin expression (Figure 2C and 2D). After suppressing endogenous NOTCH3 levels in MCF-7 cells, the morphology of MCF-7 cells was changed with obvious pseudopodia stretching out, which indicated that suppressing NOTCH3 expression might enhance the invasion and motility of cancer cells (Figure 2E).

### 3.3 miR-223 is a potential intermediate molecule for NOTCH3-suppressing ZEB1

**Figure 3:**
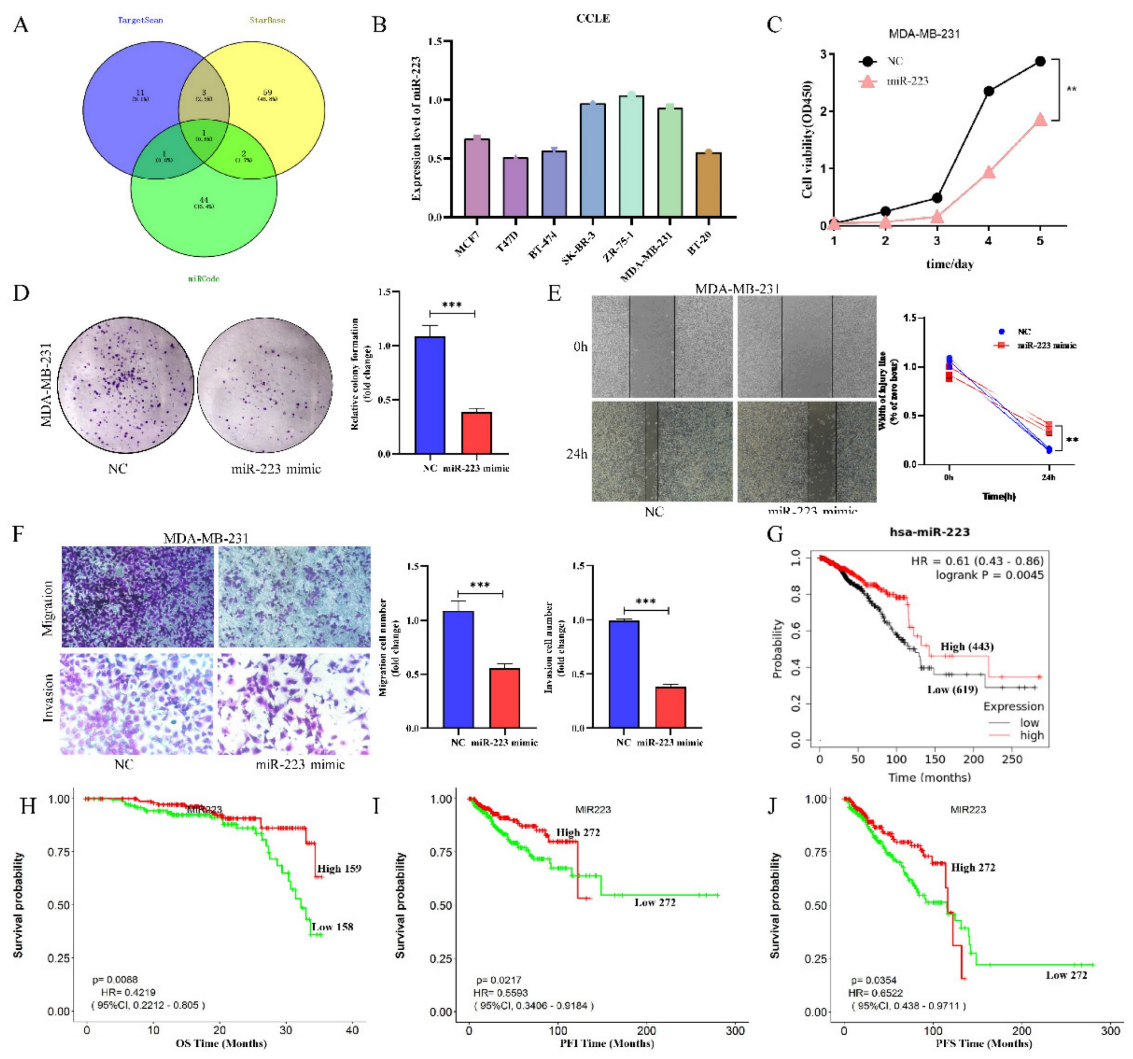
** MiR-223 is a potential intermediate regulator of NOTCH3/ZEB1, and roles as suppressor in breast cancer.** (A) Identification of miR-223 using TargetScan, StarBase and miRcode databases to search for potential miRNAs that combine with the 3'UTR of ZEB1. (B) CCLE databases showed the expression of miR-223 in different breast cancer cell lines. (C) CCK-8 assay showing the proliferation of cells was inhibited by over-expression of miR-223. (D) Colony formation of cells was inhibited by over-expression of miR-223. (E) Wound healing of cells was inhibited after over-expression of miR-223. (F) Transwell assay showing the migration and invasion of cells were inhibited by over-expression of miR-223. (G) The Kaplan-Meier Plotter database analyzes the relationship between miR-223 and the overall survival (OS) of breast cancer patients. (H/I/J) The LOGpc database analyzed the relationship between miR-223 and overall survival (OS), progression free interval (PFI) and progression free survival (PFS) of breast cancer patients.

NOTCH3 regulates downstream genes mainly through activating transcription, indicating that NOTCH3 may suppress ZEB1 expression indirectly. To identify potential intermediate molecules, TargetScan, StarBase and miRcode were used to search for potential miRNAs targeting ZEB1, and resulted in identification of one miRNA, miR-223 in common (Figure 3A). The list of potential miRNAs targeting ZEB1 is placed in Supplementary Table S2.

We further explore the expression level of miR-223 in breast cancer, online databases CCLE was mined to analyze the expression of miR-223 in different breast cancer cell lines. It was found that the expressions of miR-223 in most breast cancer cells were relatively low (Figure 3B). To investigate the function of miR-223 in breast cancer, a series of functional experiments was conducted after transfecting a miR-223 mimic into MDA-MB-231 cells. Increased miR-223 significantly suppressed cellular proliferation (Figure 3C), colony formation (Figure 3D), wound healing (Figure 3E) and invasion (Figure 3F) of TNBC cells. The prognostic value of miR-223 in breast cancer patients was also evaluated in the Kaplan-Meier Plotter and LOGpc database, finding that high expression of miR-223 was related to good prognosis of breast cancer patients (Figure 3G/H/I/J). These findings indicate that miR-223 plays an anti-cancer role in breast cancer.

### 3.4 miR-223 inhibits the proliferation and invasion of breast cancer cells via downregulating the expression of ZEB1

**Figure 4:**
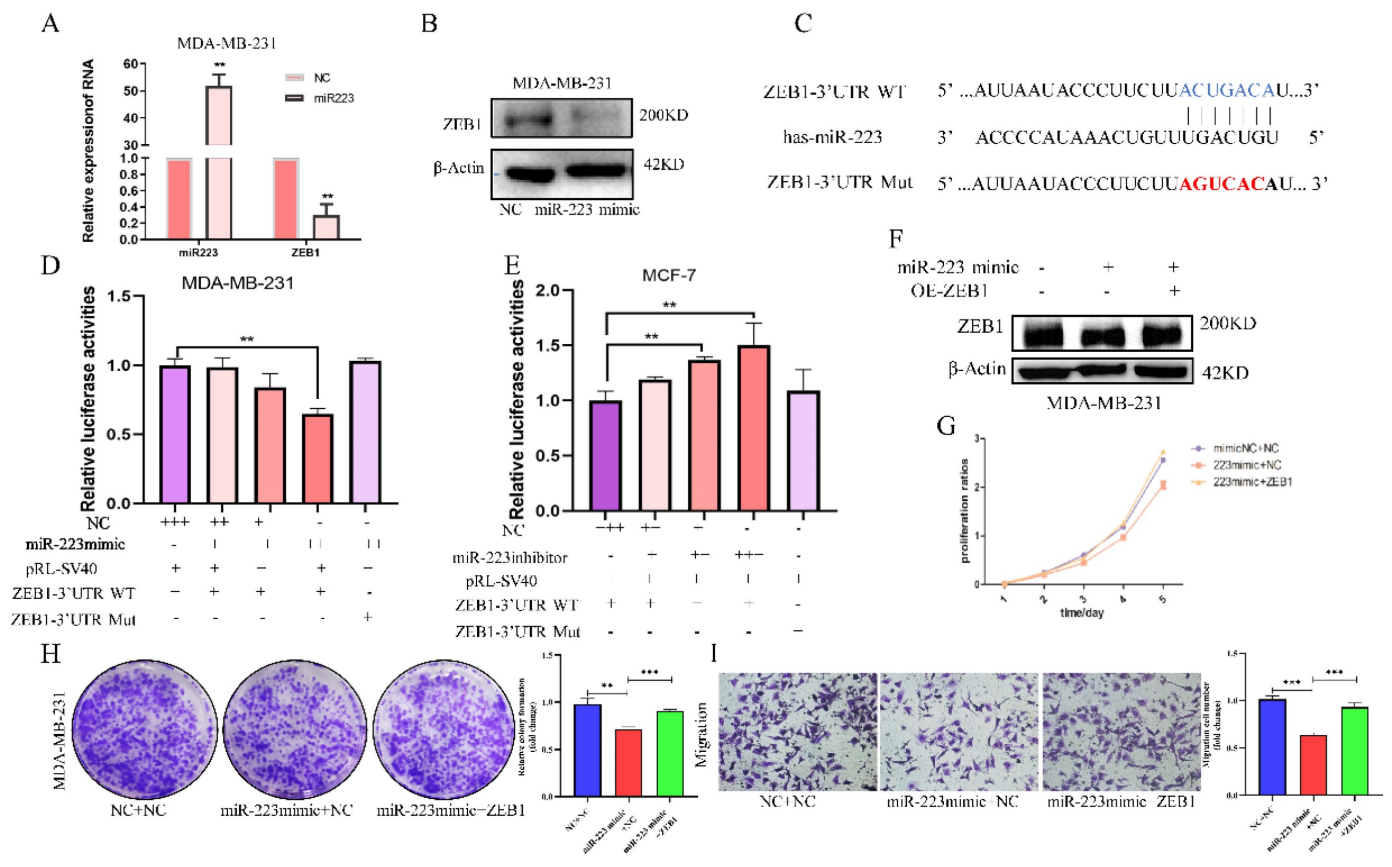
** MiR-223 inhibits the development of breast cancer cells via down-regulating ZEB1.** (A/B) PCR and western blot showing after transfecting a miR-223 mimic into MDA-MB-231 cells, the expression of ZEB1 decreased. (C) Wildtype and mutant miR-223 binding sequences in the ZEB1 3'UTR. (D/E) Reporter gene assay showing in MDA-MB-231 cells, the luciferase activity in of the ZEB1 3'UTR plasmid decreased with the dose-dependent increase of miR-223 mimic, but it did not change luciferase expression from the mutant plasmid. In MCF-7 cells, miR-223 was knocked out in a dose-dependent manner in the ZEB1 3'UTR group, and the luciferase activity increased, while the mutant group remained unchanged. (F) Western blot assay showing after miR-223 was transfected into MDA-MB-231 cells, the expression of ZEB1 decreased compared with the control group, and the expression of ZEB1 was restored after over-expression of miR-223 and ZEB1. (G/H) CCK-8 and colony formation assays showing that the inhibitory effect of miR-223 on cell proliferation can be reversed by ZEB1. (I) Migration assay showing that the inhibitory effect of miR-223 on cell migration can be reversed by ZEB1.

To verify the regulation of miR-223 on ZEB1, the effects of an miR-223 mimic, transfected into MDA-MB-231 cells, on ZEB1 was examined. ZEB1 mRNA and protein expression were both decreased by the miR-223 mimic (Figure 4A and 4B). According to the miR-223 binding sequence in the ZEB1 3'UTR, reporter plasmids, pmiRGLO-ZEB1-3'UTR and pmiRGLO-ZEB1-3'UTR-Mut were constructed to examine the ability of miR-223 to target the ZEB1 3'UTR (Figure 4C). Luciferase activity was suppressed by the miR-223 mimic in a dose-dependent manner, but it was unchanged when the mutant ZEB1 3'UTR plasmid was used (Figure 4D). On the contrary, using miR-223 inhibitor, luciferase activities were increased in a dose-dependent manner, while in the mutant group, luciferase activities remained unchanged (Figure 4E), indicating that miR-223 suppresses the expression ZEB1 by directly binding to the ZEB1 3'UTR.

Subsequently, a rescue experiment was conducted to verify the biological effect of miR-223/ZEB1. In MDA-MB-231 cells, the suppressed ZEB1 level was restored by overexpressing ZEB1 (Figure 4F). CCK-8 assay showed that the miR-223 mimic-mediated inhibitory effect on cell proliferation could be partially reversed by over-expressing ZEB1 (Figure 4G). ZEB1 also reversed the inhibitory effects of the miR-223 mimic on cell colony formation (Figure 4H) and transwell migration (Figure 4I). The above results show that, miR-223 can inhibit the proliferation and invasion of breast cancer cells via downregulating the expression of ZEB1.

### 3.5 miR-223 acts downstream of NOTCH3 in NOTCH3-mediated inhibition of breast cancer cell proliferation and invasion

**Figure 5:**
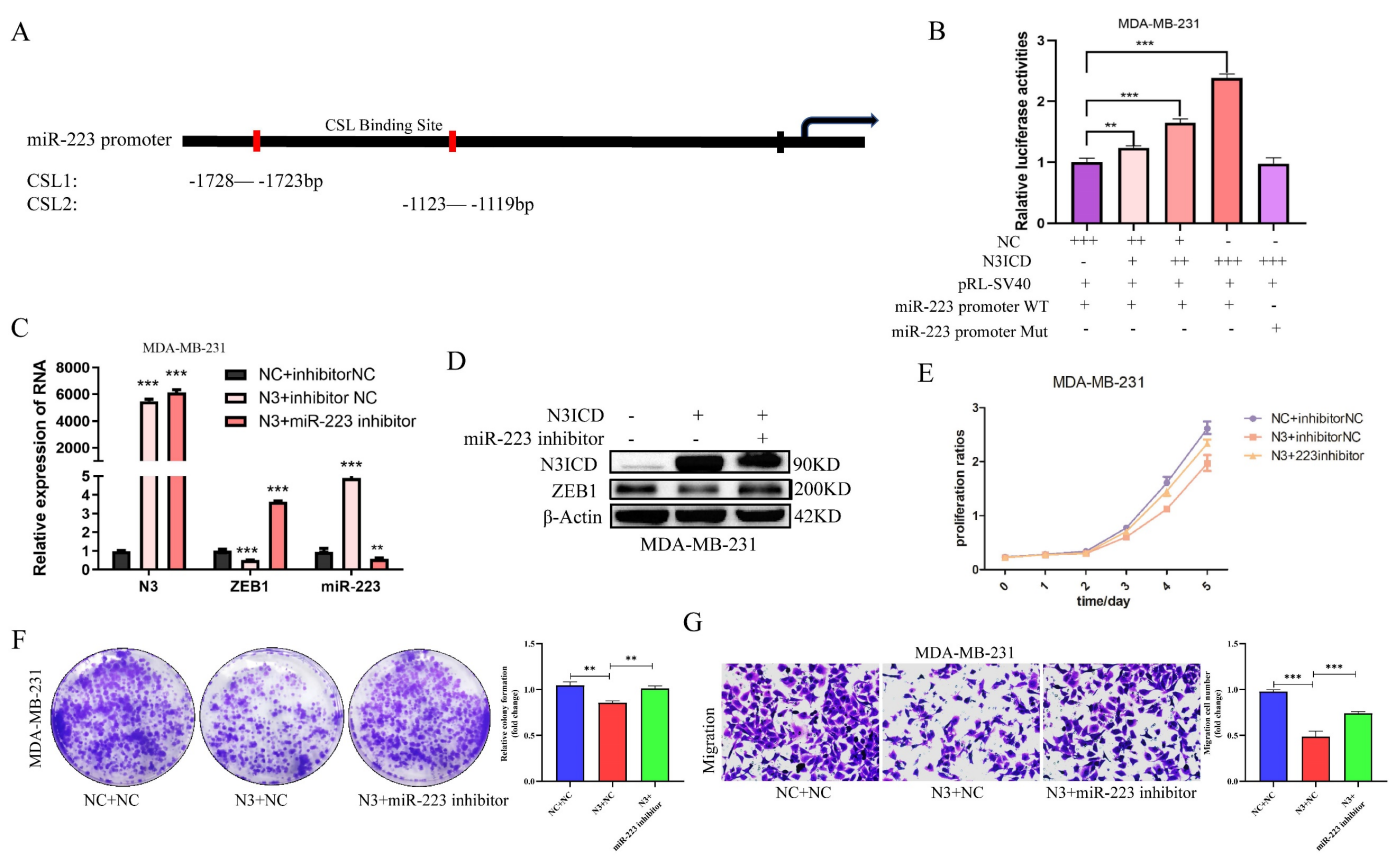
** NOTCH3 inhibits the development of breast cancer cells via up-regulating miR-223.** (A) The binding site between NOTCH3 and the promoter sequence of miR-223. (B) Reporter gene assay showing in MDA-MB-231 cells, with the increase of NOTCH3 dose-dependent pattern, the luciferase activity of miR-223 promoter-driven luciferase increased, but was unchanged in the mutant group. (C/D) Western blot and PCR assay showing after NOTCH3-transfected MDA-MB-231 cells, compared with the control group, the expression of miR-223 increased, but ZEB1 decreased. After NOTCH3 was over-expressed and miR-223 inhibitor was added, the expression levels of miR-223 and ZEB1 recovered. (E/F) CCK-8 and colony formation assays showing the inhibitory effect of NOTCH3 on cell proliferation can be reversed by miR-223 inhibitor. (G) Migration assay showing the inhibitory effect of NOTCH3 on cell migration can be reversed by miR-223 inhibitor.

It is well known that NOTCH family members directly bind to CSL promoter elements to regulate downstream target molecules. So, to explore the effect of NOTCH3 on miR-223, we searched for core CSL binding sequences (TGGGAA) in the promoter region of miR-223 and found one to be located upstream of the transcription initiation site of miR-223 (region 1: -1728 to -1723bp; region 2: -1123 to -1119 bp) (Figure 5A).

A miR-223 promoter-driven reporter gene was constructed, along with one containing a mutant CSL sequence, to examine the luciferase activities in the presence or absence of NOTCH3 intracellular segment N3ICD. In MDA-MB-231 cells, after over-expression of N3ICD, the luciferase activity driven by wild-type miR-223 promoter was increased in a dose-dependent manner, while in the mutant miR-223 promoter group, the luciferase activity did not change (Figure 5B), suggesting that NOTCH3 drives miR-223 promoter activity by directly binding to CSL binding elements.

Subsequently, rescue experiments were also carried out to verify the biological effect of NOTCH3/miR-223. In MDA-MB-231 cells, a miR-223 inhibitor was used to reverse miR-223 expression in upregulating NOTCH3 group (Figure 5C and 5D). It is found that after over-expressing NOTCH3, the expression of miR-223 was up-regulated, and the mRNA and protein levels of ZEB1 were decreased. Adding a miR-223 inhibitor decreased the expression of miR-223 and concomitant with upregulation of ZEB1 expression.

CCK-8 assay showed that the inhibitory effect of NOTCH3 over-expression on cell proliferation was partially reversed by the miR-223 inhibitor (Figure 5E). Colony formation assays showed that inhibiting the expression of miR-223 reversed the inhibitory effect of NOTCH3 on cell colony formation (Figure 5F). Similar results were also found for migration (Figure 5G). These results provide further support that NOTCH3 inhibits the proliferation and invasion of breast cancer cells by up-regulating the expression of miR-223.

### 3.6 Prognostic value of NOTCH3 and ZEB1 in breast cancer

**Figure 6:**
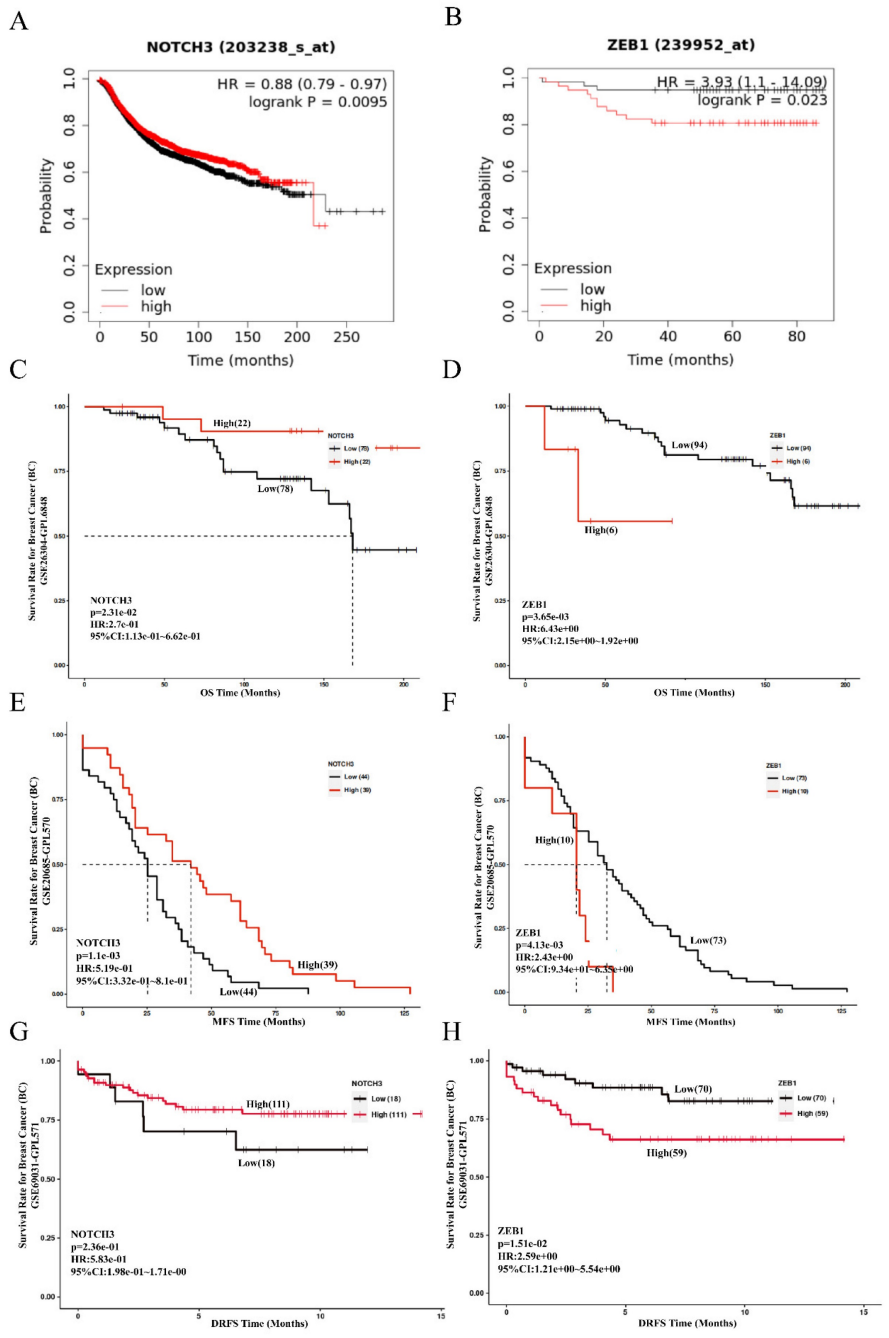
** Prognostic value of NOTCH3 and ZEB1 in breast cancer.** (A/B/C/D) Kaplan-Meier Plotter and PanCanSurvPlot showing high expression of NOTCH3, as well as the low expression of ZEB1, were related to good OS of breast cancer patients. (E/F) PanCanSurvPlot showing high expression of NOTCH3, as well as low expression of ZEB1, were related to good MFS of breast cancer patients. (G/H) PanCanSurvPlot showing high expression of NOTCH3, as well as low expression of ZEB1, were related to good DRFS of breast cancer patients.

To evaluate the role of NOTCH3/ZEB1 in patients with breast cancer, the prognostic value of NOTCH3 and ZEB1 was examined in the Kaplan-Meier Plotter and GEO databases (GSE19615) using the default median method to define low or high groups. A low level of NOTCH3 or high level of ZEB1 was correlated with poor overall survival (OS) of patients with breast cancer, with HR = 0.88, *p* = 0.0095 and HR = 3.93, *p* = 0.023, respectively (Figure 6A and 6B).

The results obtained from the PanCanSurvPlot were consistent. The define of different groups was based on the optimal (maximally selected rank statistics) method to define low or high groups) were consistent. In the GSE26304 dataset 35, NOTCH3 expression was positively correlated with OS (*p* = 0.0231, HR = 0.274) (Figure 6C), whereas ZEB1 expression was negatively associated with OS of breast cancer patients (*p* = 0.00365, HR = 6.43) (Figure 6D). As metastasis is the principal feature of malignancies that affects the prognosis of patients, the GSE20685 dataset 36 was also recruited in this study to evaluate the role of NOTCH3 and ZEB1. Consistently, for breast cancer patients, higher expression of NOTCH3 was positively correlated with metastasis-free survival (MFS) (*p* = 0.0011, HR = 0.519) (Figure 6E), whereas the expression level of ZEB1 was negatively correlated with MFS (*p* = 0.00413, HR = 2.43) (Figure 6F). To assess their role in predicting relapse of breast cancer, the GSE69031 dataset with distant relapse-free survival (DRFS) information was applied. Breast cancer patients with high expression of NOTCH3 tended to have long DRFS (*p* = 0.236, HR = 0.583) (Figure 6G), while high ZEB1 levels predicted poor DRFS in patients with breast cancer (p = 0.0151, HR = 2.59) (Figure 6H).

## 4. Discussion

We show that NOTCH3 up-regulates the expression of miR-223 by directly binding to the CSL core element in its promoter, and miR-223 inhibits the translation of ZEB1 by directly binding to the 3' UTR region of ZEB1, which eventually leads to the proliferation, invasion and EMT inhibition of breast cancer cells.

Our results confirm the negative-correlation of NOTCH3 with EMT. NOTCH3 is down-regulated in TGF-β-induced breast cancer cell MCF-7, consistent with previous reports. Wei *et al.* found that NOTCH3 inhibits EMT by inhibiting Bmi1, and up-regulates estrogen receptor (ER)α in breast cancer 37. Interestingly, transcription factor ZEB1 is up-regulated in TGF-β-induced MCF-7 cells. The expressions of NOTCH3 and ZEB1 are negatively correlated in breast cancer. Moreover, the enhanced expression of NOTCH3 leads to the down-regulation of ZEB1 in TNBC cells, while the inhibition of NOTCH3 expression in MCF-7 cells leads to the up-regulation of ZEB1. This shows that NOTCH3 can negatively regulate the expression of ZEB1.

NOTCH3 activates downstream target genes by directly binding to CSL promoter elements. For example, in breast cancer, NOTCH3 trans-activates PTEN by binding a CSL binding element in PTEN promoter 38. NOTCH3 promotes adipocyte differentiation of 3T3-L1 preadipocytes by directly up-regulating LARS expression and activating the mTOR pathway 39. That indicates that NOTCH3 may not directly negatively regulate the expression of ZEB1.

Many studies have shown that non-coding small ribonucleic acid (miRNA) inhibits the translation of target genes or promotes target gene degradation by binding to the 3' untranslated region (3'-UTR) of target mRNA 40. Through data mining, we identified miR-223 as a potential miRNA that regulates the expression of ZEB1. MiRNA-223 was first reported in 2005 41, it is located on q12 of the X chromosome, and is mainly observed in cells of bone marrow lineage, especially neutrophils 42, 43. MiR-223 has been reported in many cancers, including breast cancer 44. Although it is reported that miR-223 is highly expressed in breast cancer and coordinates breast cancer progression 45, 46, most studies only focused on the expression pattern of miR-223, and found that the expression of miR-223 in luminal and HER2 subtypes of breast cancer is decreased, and high expression of miRNA-223 is an indicator of good prognosis in TNBC 47-49. Over-expression of miR-223 in breast cancer cells leads to a decrease in cell proliferation, migration and invasion. MiR-223 inhibits protein translation of ZEB1 by binding to the 3'UTR of ZEB1 mRNA in different cells. In bladder cancer cells, miR-223-3p can inhibit protein translation of ZEB1 50. MiR-223 can also directly inhibit the expression of ZEB1 to promote the differentiation of skeletal myoblasts 33. In addition, miR-223 can increase the radiosensitivity of nasopharyngeal carcinoma cells by directly inhibiting the expression of ZEB1 51. However, the regulatory effect of miR-223 on ZEB1 has not been reported in breast cancer. Our results show that miR-223 can regulate the expression of ZEB1 in breast cancer cells. Using reporter gene assays, miR-223 can directly bind to the 3'UTR of ZEB1 in breast cancer cells, indicating that miR-223 directly inhibits the expression of ZEB1. Moreover, in breast cancer cells, miR-223 inhibits the proliferation and migration of cells by inhibiting the expression of ZEB1.

As previously reported, NOTCH3 stimulates the expression of target genes by binding to the promoter element of the downstream target gene. CSL binding elements were recognized by NOTCH3 in the promoter region of miR-223 through prediction, and NOTCH3 can activate the miR-223 promoter and up-regulate the expression of miR-223 in T-cell acute lymphoblastic leukemia 52. In addition, NOTCH3 can regulate the expression of miR-223 and increase the production of cytokines in macrophages of patients with rheumatoid arthritis 53. In reporter gene assays, NOTCH3 directly binds to the promoter element of miR-223 in breast cancer cells, which indicates that NOTCH3 directly promotes the expression of miR-223. Similarly, our results also show that NOTCH3 inhibits cell proliferation and migration by promoting the expression of miR-223.

In patients with breast cancer, the level of NOTCH3/miR-223/ZEB1 was analyzed and their prognostic value determined. The results showed that high expression of miR-223 and NOTCH3 are related to good prognosis of breast cancer patients. On the contrary, high expression of ZEB1 is related to poor prognosis of breast cancer patients. These results support the discovery that NOTCH3, miR-223 and ZEB1 may be potential biomarkers of breast cancer.

Previously, it has been reported that the expression of NOTCH3 is positively correlated with a lower Ki-67 index and the incidence of involved lymph node status in breast cancer patients, and predicts a better recurrence-free survival rate of breast cancer patients 38. In addition, NOTCH3 up-regulates the expression of GSK3β, which is related to better recurrence-free survival rate of all breast cancer patients 14. NOTCH3 is very weakly expressed in breast cancer cells, which prevents tumor occurrence through HeyL-mediated inhibition of Mybl2 (an important cell cycle regulator), and is related to good prognosis 15. It should be noted that the effect of NOTCH3 in breast cancer has also been reported. About one third of TNBC is related to the amplification or over-expression of NOTCH3 and an over-activated NOTCH3 signal, which may be caused by a different tumor microenvironment 54, 55. As for miR-223, it has been observed that the level of miR-223 decreases during the transition from healthy breast tissue, ductal carcinoma in situ and invasive ductal carcinoma, suggesting that miR-223 could be used as a marker to identify the progress of cancer 47. In the samples collected from breast cancer patients before and after operation, it was found that circulating miR-223 level decreased after operation 56. However, it is worth noting that the level of miR-223-3p in patients with invasive ductal carcinoma is higher than that in patients with ductal carcinoma in situ, suggesting that use of miR-223 as a biomarker in these cases is still controversial 57. There have been many reports about the high expression of ZEB1 and the poor prognosis of breast cancer patients 58. It is reported that YEATS4 regulates the expression of ZEB1 in breast cancer, promotes EMT and metastasis, and is related to poor prognosis 59. In addition, the existence of the ZEB1/KLF5-mTOR-CCND1/ABCB1 axis may be involved in the paclitaxel response pathway, and it is influencing the susceptibility and prognosis of breast cancer 60. These studies show that there is a negative correlation between expression and function between NOTCH3 and ZEB1 in breast cancer. NOTCH3 may inhibit the progress of breast cancer through a multi-layer system, and it may at least partially inhibit the expression of ZEB1 and inhibit the proliferation, migration and EMT in breast cancer cells by inducing miR-223. However, further experimental design and research are needed to verify the effect of NOTCH3/miR-223/ZEB1 axis on breast cancer at the animal and clinical levels.

Notably, a direct inhibitory effect of ZEB1 on NOTCH3 expression has been reported in squamous cell carcinoma 61. In fact, there are negative regulatory loops in ZEB1, such as a ZEB1/miR-200 feedback loop. The ZEB1 3'UTR contains eight binding sites for miR-200 62, and many studies have described ZEB1 as a key target of miR-200 family members 63, 64. Interestingly, knocking out ZEB1 led to an increase in the expression of all miR-200 family members 65. In breast cancer, there may be such a negative regulatory loop between ZEB1 and NOTCH. However, this remains to be answered and needs more rigorous experiments for verification. This investigation further describes the possible role of NOTCH3/miR-223/ZEB1 in breast cancer. It provides new insight into the complex regulation of breast cancer, and a basis for further mining the potential candidates for breast cancer prognosis indicators and/or treatment approaches.

## Supplementary Material

Supplementary figure and tables.

## Figures and Tables

**Table 1:** siRNA sequence targeting Notch3 and ZEB1

Genes	Sequences (5' to 3')
siNC	UUCUCCGAACGUGUCACGUTT
siNotch3-1	GAGCCAAUAAGGACAUGCA
si Notch3-2	UAUAGGUGUUGACGCCAUCCACGCA
siZEB1-1	GCCCUAUCCCUUUACGUCA
siZEB1-2	CCUAGUCAGCCACCUUUAATT

**Table 2:** Primer sequences for qRT-PCR

Primer	Sequence (5'to3')	Production (bp)
Notch3	F ATGCAGGATAGCAAGGAGGA	180
Notch3	R AAGTGGTCCAACAGCAGCTT	
ZEB1	F ACCTCTTCACAGGTTGCTCCT	200
ZEB1	R AGTGCAGGAGCTGAGAGTCA	
β-actin	F GGGAAATCGTGCGTGACATTAAG	128
β-actin	R TGTGTTGGCGTACAGGTCTTTG	
hsa-miR-223	F TGACGGCGTGTATTTGACAAG	
hsa-miR-223	R TATGGTTGTTCTCGACTCCTTCAC	

**Table 3:** Antibody information

Antibody	Company	Source	KDa	Article number	Dilution rate
Notch3	CST	Rabbit	270, 90	#5276	1:1000
E-cadherin	CST	Rabbit	110	#3195	1:2000
Vimentin	CST	Rabbit	57	#5741	1:1000
ZEB1	CST	Rabbit	200	#3396	1:500
β-actin	Santa	Mouse	42	TA-09	1:3000
