# Supplementary material for: NOTCH3 inhibits transcription factor ZEB1 expression and metastasis of breast cancer cells via transcriptionally upregulating miR-223
Source: J Cancer. 2024 Jan 1;15(1):192–203. doi: 10.7150/jca.89034 (PMC10751662; doi:10.7150/jca.89034)
Supplement: Supplementary file 1 — Supplementary figure and tables. [file jcav15p0192s1.pdf]

Supplementary materials

Supplementary Figures

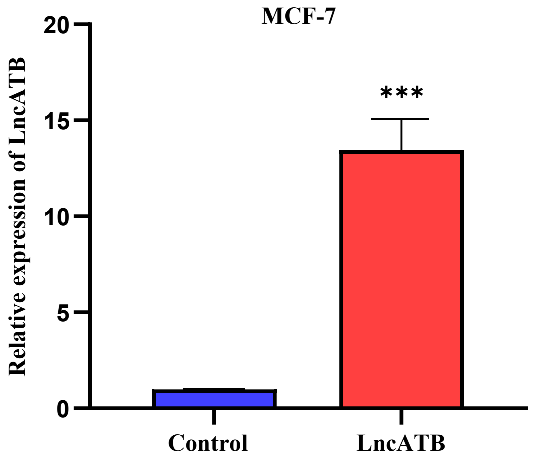

Supplementary Figure S1: After overexpressing lncATB in MCF-7 cells, the overexpression efficiency of lncATB was detected by qRT-PCR. The relative expression levels of lncATB was calculated and normalized relative to β-actin using the 2-ΔΔCt method.

Supplementary Tables

Supplementary Table S1. Primers used for examining the expression of lncATB

Primer names	Sequences
lncATB sense	5'-TCTGGCTGAGGCTGGTTGAC-3'
lncATB anti-sense	5'-ATCTCTGGGTGCTGGTGAAGG-3'

Supplementary Table S2. The list of predicted miRNAs targeting ZEB1

Datasets	Predicted miRNAs targeting ZEB1
TargetScan	hsa-miR-96-5p, hsa-miR-506-3p, hsa-miR-454-3p, hsa-miR-429, hsa-miR-301-3p, hsa-miR-223-3p, hsa-miR-200bc-3p, hsa-miR-200a-3p, hsa-miR-199-3p, hsa-miR-183-5p, hsa-miR-142-3p, hsa-miR-141-3p, hsa-miR-130-3p, hsa-miR-1271-5p, hsa-miR-124-3p.2<br>hsa-miR-101-3p.1
StarBase	hsa-miR-524-5p, hsa-miR-520d-5p, hsa-miR-519c-3p, hsa-miR-519b-3p, hsa-miR-495-3p, hsa-miR-494-3p, hsa-miR-452-5p, hsa-miR-448, hsa-miR-433-3p, hsa-miR-431-5p, hsa-miR-429, hsa-miR-410-3p, hsa-miR-409-3p, hsa-miR-382-5p, hsa-miR-381-3p, hsa-miR-374a-5p, hsa-miR-369-3p, hsa-miR-365a-3p, hsa-miR-361-5p, hsa-miR-34c-5p, hsa-miR-342-3p, hsa-miR-33a-5p<br>hsa-miR-326, hsa-miR-32-5p, hsa-miR-323a-3p, hsa-miR-301a-3p, hsa-miR-27b-3p, hsa-miR-27a-3p, hsa-miR-23b-3p, hsa-miR-23a-3p, hsa-miR-223-3p<br>hsa-miR-219a-5p, hsa-miR-217, hsa-miR-216a-5p, hsa-miR-211-5p, hsa-miR-208a-3p, hsa-miR-205-5p, hsa-miR-204-5p, hsa-miR-200c-3p, hsa-miR-200b-3p, hsa-miR-200a-3p, hsa-miR-199a-3p, hsa-miR-196b-5p, hsa-miR-194-5p<br>hsa-miR-186-5p, hsa-miR-182-5p, hsa-miR-181d-5p, hsa-miR-181c-5p, hsa-miR-181b-5p, hsa-miR-181a-5p, hsa-miR-154-5p, hsa-miR-153-3p, hsa-miR-147a, hsa-miR-146b-5p, hsa-miR-146a-5p, hsa-miR-144-3p, hsa-miR-143-3p<br>hsa-miR-141-3p, hsa-miR-140-5p, hsa-miR-139-5p, hsa-miR-130b-3p, hsa-miR-130a-3p, hsa-miR-128-3p, hsa-miR-124-3p, hsa-miR-105-5p
miRcode	hsa-miR-96, hsa-miR-93, hsa-miR-9, hsa-miR-876-3p, hsa-miR-7, hsa-miR-520, hsa-miR-519a, hsa-miR-5127, hsa-miR-507, hsa-miR-497, hsa-miR-4770, hsa-miR-428, hsa-miR-373, hsa-miR-372, hsa-miR-322, hsa-miR-302abcde, hsa-miR-295, hsa-miR-294, hsa-miR-291a-3p, hsa-miR-223-3p, hsa-miR-212, hsa-miR-200a, hsa-miR-195, hsa-miR-194-5p, hsa-miR-16, hsa-miR-15abc, hsa-miR-155, hsa-miR-153, hsa-miR-152, hsa-miR-150, hsa-miR-148ab-3p, hsa-miR-146, hsa-miR-144, hsa-miR-143, hsa-miR-142-3p, hsa-miR-1420ac, hsa-miR-141, hsa-miR-140, hsa-miR-139-5p, hsa-miR-138, hsa-miR-1378, hsa-miR-137, hsa-miR-135, hsa-miR-132, hsa-miR-1271, hsa-miR-1244, hsa-miR-106a, hsa-miR-105
